# The Anesthetic Management of a Parturient With Osteogenesis Imperfecta Type I Undergoing Cesarean Delivery

**DOI:** 10.7759/cureus.13849

**Published:** 2021-03-12

**Authors:** Manshu Yan, Nicholas P Knowland, Donna Lien

**Affiliations:** 1 Anesthesiology and Perioperative Medicine, Loma Linda University Medical Center, Loma Linda, USA

**Keywords:** osteogenesis imperfecta, anesthetic concern, caesarean delivery, spinal anesthesia

## Abstract

Osteogenesis imperfecta (OI) is a rare disorder of bone fragility caused by mutations in the COL1A1/2 genes, which encode type I procollagen. It commonly manifests with bone fractures, joint dislocations, and easy bruising. OI patients presenting for surgery may pose multiple challenges to the anesthesiologist such as management of a potentially difficult airway and heightened positional fracture risks. We present a case detailing the spinal anesthetic management of a 28-year-old woman with type I OI requiring cesarean delivery for a 32-week intrauterine pregnancy with fetal cardiac anomalies.

## Introduction

Osteogenesis imperfecta (OI) affects approximately 1 in 10,000 to 20,000 people worldwide with an estimation of 25,000 to 50,000 people in the United States being affected. The disease encompasses a group of genetic disorders encoding mutations in genes affecting type I collagen. Inherited autosomal dominant mutations in the COL1A1/2 genes cause approximately 90% of all cases of OI, while the rest can be autosomal recessive or X-linked [1]. OI is classified into five subgroups based on clinical symptoms and severity, including classic non-deforming OI with blue sclerae (OI type I), perinatally lethal OI (OI type II), progressively deforming OI (OI type III), common variable OI with normal sclerae (OI type IV) and OI with calcification in interosseous membranes (OI type V). The severity grading scale has been proposed according to clinical, fracture frequency, bone densitometry, and level of mobility [2].

We present and discuss anesthetic concerns in the management of a gravid patient with type I OI presenting for cesarean section (c-section). Concerns included positioning to mitigate fracture risk, a technically challenging spinal anesthetic placement in the patient with vertebral compression fracture, and hearing loss associated with opioid use. The patient provided written Health Insurance Portability and Accountability Act (HIPAA) authorization for the publication of this case report.

## Case presentation

A 28-year-old multiparous woman at 32 weeks of gestation was admitted for fetal monitoring of fetal intrauterine growth restriction (IUGR) and cardiac abnormalities including fetal aortic regurgitation, right ventricular (RV) dilation, tricuspid regurgitation, low normal RV function, and umbilical arterial flow reversal on Doppler. The anesthesiology team was consulted in anticipation of a possible surgical delivery. 

**Figure 1:**
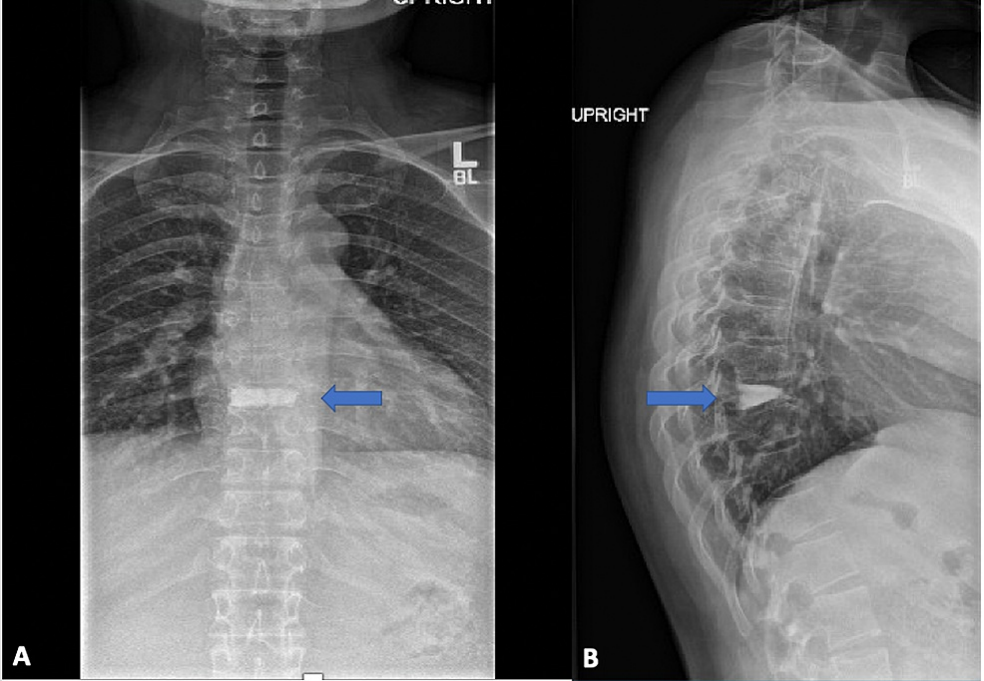
X-ray thoracic spine anteroposterior (AP) and lateral view A, X-ray thoracic spine AP view showing T9 kyphoplasty (blue arrow); B, X-ray thoracic spine lateral view showing T9 kyphoplasty (blue arrow)

**Figure 2:**
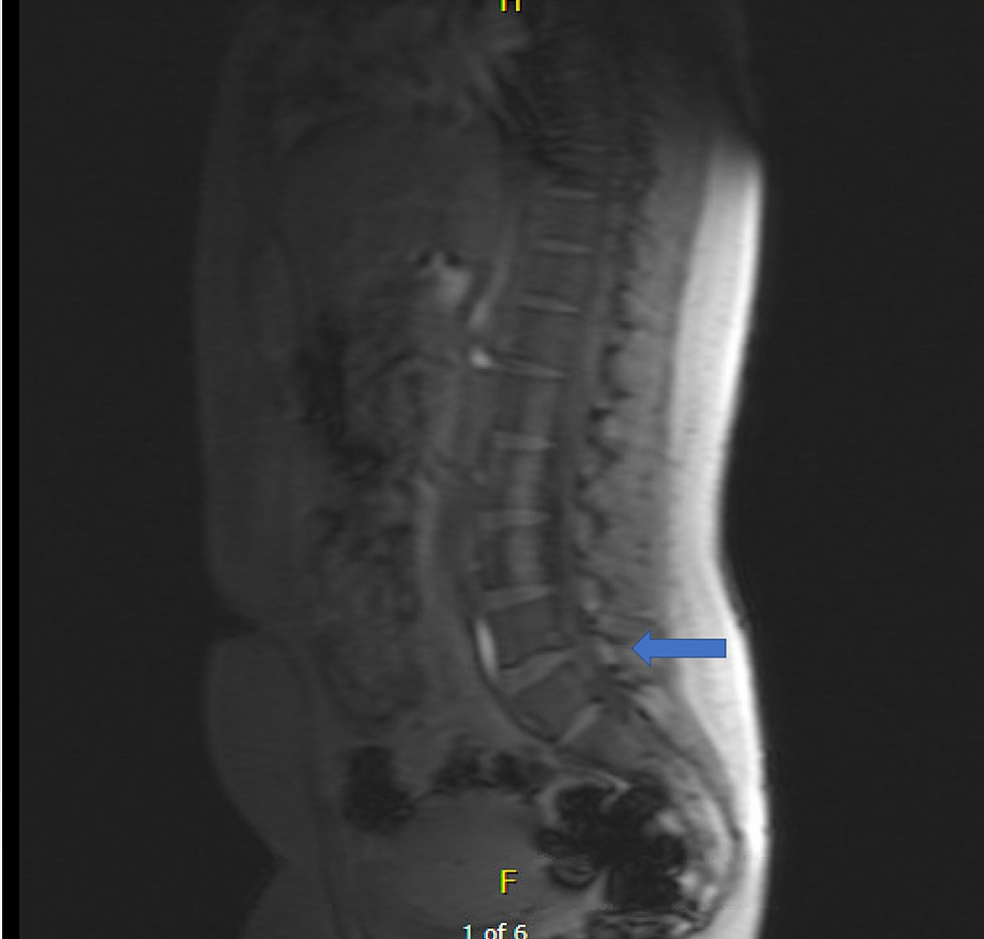
MRI lumbar spine sagittal view MRI lumbar spine sagittal view showing L4-5 disk degeneration with mild spinal stenosis (blue arrow)

The patient’s past medical history included type I OI confirmed by genetic testing with COL1A1/2 gene mutation, multiple related fractures including bilateral wrist fractures, ankle fracture and a ninth thoracic vertebrae (T9) compression fracture and underwent kyphoplasty one year prior, well-controlled gestational diabetes, and two prior uncomplicated vaginal deliveries with patient-controlled epidural analgesia (PCEA). Family history was significant for OI in two sons and multiple siblings. Physical exam identified short stature of 149 centimeters, weight of 68 kilograms, blue sclerae, and mild scoliosis. Airway exam demonstrated Mallampati class II with normal mouth opening, upper and lower dentures, normal range of neck motion, and a thyromental distance more than 6.5 cm. Lab results including hematology and coagulation studies were within normal limits. A review of the past spine X-ray identified the T9 compression deformity with kyphoplasty (Figure 1), lumbar MRI showed fourth and fifth lumbar (L4-5) disk degeneration with mild spinal stenosis and osteopenia (Figure 2). Chest X-ray and echocardiogram were normal. Benefits and risks of a spinal anesthetic with possible general anesthesia were explained to the patient. The patient raised concern about opioids, informing the team that intravenous (IV) fentanyl had previously led to hearing loss lasting for an hour. The patient agreed to the plan provided. Efforts would be made to avoid the use of opioids, if possible.

On hospital day two, 24 hours after betamethasone administration to promote fetal lung maturity, fetal heart rate (FHR) monitoring demonstrated intermittent and variable decelerations. The decision was made to proceed with a primary c-section and intrauterine device placement. In the operating room, an 18G IV was placed with intravenous (IV) crystalloid attached to the patient, the patient was placed on standard American Society of Anesthesiologists (ASA) monitors with the blood pressure (BP) cuff set to cycle every five minutes. The patient was then seated upright on the bed with forward flexion to optimize the spinal anesthetic placement. The L4-5 interspace was palpated and a 24-gauge 3.5-inch pencil-point needle was inserted, with interval assessment for entrance into the intrathecal space. On the second attempt free-flowing cerebrospinal fluid (CSF) was identified and 13.5 milligrams (mg) of 0.75% hyperbaric bupivacaine was injected intrathecally with no opioid or epinephrine addition. The patient was then positioned supine with a 15-degree left tilt to avoid aortocaval syndrome. The extremities were padded with care to avoid bony trauma or pressure injury. Cold alcohol swab tests at three and five minutes demonstrated anesthesia level to the T4 dermatome. Phenylephrine infusion was started at 50 micrograms (mcg)/minute and titrated to maintain systolic blood pressure within 20% of normal range according to our institutional protocol. Patient's hemodynamics remained stable throughout the case. 

Cesarean delivery was performed via a mid-transverse uterine incision given fetus IUGR with delivery of a 1,360-gram male baby who was transferred to the neonatal intensive care unit team. Appearance, pulse, grimace, activity and respiration (APGAR) scores were 6 at one minute and 9 at five minutes. After delivery, the mother was given a bolus of 3 units (U) of oxytocin IV and a slow 40 U oxytocin/1 liter normal saline infusion was started to maintain uterine tone. One hour after spinal anesthetic administration, the patient experienced sharp breakthrough pain while the surgeon was suturing the uterine wall. Pain was initially treated with 50 mcg of IV fentanyl. Immediately the patient started having hearing difficulties. Pain remained uncontrolled and patient was further treated with a 10 mg ketamine bolus. A propofol infusion was started at 30 mcg/kg/minute for sedation. Further opioid treatment was not attempted. In the following hour, an additional 20 mg IV ketamine and 50 mg IV propofol were given for analgesia and sedation. At the end of the procedure, 30 mg IV ketorolac, 20 mcg IV dexmedetomidine, and 1000 mg IV acetaminophen were given for multimodal pain control. The patient was transferred to the post-anesthetic care unit (PACU) without further complications. The patient’s hearing fully returned in PACU. Post-operative pain was sufficiently controlled with a combination of acetaminophen, methocarbamol, ibuprofen, and gabapentin. On post-operative day four, the patient was discharged home without complication; the baby remained under NICU care. 

## Discussion

Osteogenesis imperfecta is a clinically heterogeneous heritable connective tissue disorder. Bone fragility, short stature, scoliosis, macrocephaly, conductive deafness, or odontological deformities are common. OI has various extra musculoskeletal systemic manifestations. Neurological manifestations may include hydrocephalus, syringomyelia, and basilar invagination. Restrictive lung disease can be caused by thoracic skeletal deformities which may lead to recurrent pneumonia, progressing to pulmonary hypertension or right-sided heart failure. Cardiovascular findings such as valvular insufficiency, aortic root dilatation, atrial septal defects and septal/posterior left ventricular wall thickening have also been reported in OI. Complications from the respiratory and cardiovascular systems are the most common causes of OI-related mortality [2]. 

Medical management of OI typically includes both physical therapy and pharmacologic therapy with bisphosphonates and growth hormone to promote increased strength, mobility, and linear bone growth [3]. Surgery is also a mainstay of lifelong OI management for fracture prophylaxis/treatment with intramedullary rods or correction of scoliosis [4]. 

Fertility is typically preserved in patients with OI and pregnancy can be carried to term if there are no other complications. Musculoskeletal-related complications range from mild back pain to vertebral compression fractures, disc and ligament problems [5]. Respiratory compromise and small body habitus may necessitate preterm delivery in 27% of cases [6]. Delivery by c-section is common; one study found a breech presentation rate of 32% and a c-section rate of 54% in OI patients. Of these c-sections, 53% were for non-vertex presentation and 15% for antenatal diagnosis of OI [6]. 

Patients with OI pose significant challenges for the anesthesiologist. Bone frangibility and malformation can increase the risk for fractures and dislocations related to positioning or even with BP cuff inflation. Thus, pressure points should be well-padded and arterial line placement is recommended in patients with severe OI undergoing a prolonged procedure, although some studies dispute this later recommendation [7]. OI patients are prone to mandibular fractures, odonto-axial dislocations, and teeth injury due to dentinogenesis imperfecta and also have an increased risk for a difficult airway. Patients should be carefully evaluated for a history of difficult airway/mask ventilation and cervical spine instability. Successful intubations with video laryngoscope, fiberoptic scope and through a laryngeal mask airway have been reported [8,9]. The safety of regional anesthesia has been described in both case reports and small case series detailing successful spinal, epidural, and nerve block techniques in OI patients [10]. Spinal deformities can make spinal or epidural placement challenging or lead to uneven sensory blockade. OI patients exhibit an increased risk of hyperthermia under general anesthesia but studies have only found a weak association between OI and malignant hyperthermia [11]. Nevertheless, succinylcholine should be avoided given the increased risk for contracture-induced fractures and hyperthermia. Patients with OI often have associated low platelet counts, reduced factor VIII activity, increased capillary fragility, and impaired platelet aggregation leading to increased bleeding risks. Prior to regional procedures a platelet count and coagulation studies should be obtained, and blood cross-matching can be necessary to ensure rapid treatment of potential bleeding. Finally, the anesthetic plan needs to consider optimizing ventilation in OI patients with significant restrictive lung disease, regional anesthesia is preferred to general anesthesia for the condition, if general anesthesia is required then a low tidal volume, higher frequency, pressure control ventilation strategy is preferred. 

In this case the patient reported severe hearing loss as a side-effect of fentanyl administration. In OI, hearing loss is typically conductive in young patients and sensorineural in older patients. The prevalence increases with age and incidence is highest in type I OI [12]. Up to a 95% rate of hearing loss has been reported in type I OI patients over 30 years of age [13]. Theoretically, conductive hearing loss is due to stapes fixation and sensorineural loss is related to the atrophy of cochlear hair cells [14]. In recent years, several reports have emerged describing opioid-induced sudden sensorineural hearing loss, suggesting that opioids affect the inner ear through undetermined mechanisms [15]. Additionally, clinical or subclinical hearing loss following spinal anesthesia is a known side-effect, manifesting as a low-frequency hearing deficit, perhaps related to CSF leakage precipitating a pressure imbalance of the inner ear. The mechanism of hearing loss after fentanyl dosage in this patient is unclear but could be the result of any of the above effects or to their combination. The duration of the patient’s spinal anesthetic with bupivacaine was shorter than expected (60 minutes), which could be contributed to the lack of addition of opioid to the injection, or spinal deformity/scar tissue caused by patient’s previous spine injury and spinal stenosis. In hindsight, epinephrine could have been added to 0.75% bupivacaine to increase the duration of anesthetic effect, or an alternative combined spinal epidural (CSE) anesthesia could have been performed. 

## Conclusions

In conclusion, osteogenesis imperfecta is a rare disorder and the patient population frequently requires surgical and therefore anesthesia management. The peri-operative risks and potential complications are increased in OI patients, which include fracture and dislocation, difficult airway, difficult/unpredictable regional anesthesia, cardiopulmonary complications, and bleeding disorder, etc. OI patients need careful peri-operative anesthetic management to optimize care.
